# Predicting Psychological Distress Amid the COVID-19 Pandemic by Machine Learning: Discrimination and Coping Mechanisms of Korean Immigrants in the U.S.

**DOI:** 10.3390/ijerph17176057

**Published:** 2020-08-20

**Authors:** Shinwoo Choi, Joo Young Hong, Yong Je Kim, Hyejoon Park

**Affiliations:** 1 School of Social Work, Texas State University, San Marcos, TX 78666, USA; 2 Department of Exceptional, Deaf, and Interpreter Education, University of North Florida, Jacksonville, FL 32224, USA; jooyoung.hong@unf.edu; 3 Ingram School of Engineering, Texas State University, San Marcos, TX 78666, USA; 4 Department of History, Philosophy, and Social Sciences, Pittsburg State University, Pittsburg, KS 66762, USA; hyejoon.park@pittstate.edu

**Keywords:** COVID-19, racism, mental health, Korean immigrants, United States, Artificial Neural Network

## Abstract

The current study examined the predictive ability of discrimination-related variables, coping mechanisms, and sociodemographic factors on the psychological distress level of Korean immigrants in the U.S. amid the COVID-19 pandemic. Korean immigrants (both foreign-born and U.S.-born) in the U.S. above the age of 18 were invited to participate in an online survey through purposive sampling. In order to verify the variables predicting the level of psychological distress on the final sample from 42 states (*n* = 790), the Artificial Neural Network (ANN) analysis, which is able to examine complex non-linear interactions among variables, was conducted. The most critical predicting variables in the neural network were a person’s resilience, experiences of everyday discrimination, and perception that racial discrimination toward Asians has increased in the U.S. since the beginning of the COVID-19 pandemic.

## 1. Introduction

First reported in December 2019, the number of coronavirus disease 19 (COVID-19) cases has rapidly increased, threatening global public health [1]. As of 6 July 2020, there were more than 11 million confirmed cases and 532,340 confirmed deaths in 216 countries, according to the World Health Organization (WHO), which declared an international public emergency as the result of the COVID-19 outbreak [2]. In the U.S., the first confirmed case was reported in late January. As of 6 July 2020, nearly 3 million confirmed cases and about 130,000 deaths were identified, representing the most significant number of confirmed cases and deaths worldwide [3]. Presently, the impact of the COVID-19 pandemic is tremendous throughout the world, causing a considerable amount of pain in every aspect of the global society. The extent of the damage to our community is unmeasurable and unpredictable, as the pandemic is still fast-growing. The objective of the current study was to explore the effects of discrimination and coping mechanisms on Korean immigrants’ psychological distress amid the COVID-19 pandemic by using Artificial Neutral Network (ANN) modeling.

### 1.1. Korean Immigrant Population in the U.S.

Among the various Asian groups in the U.S., Korean American is the fifth largest subgroup of the diverse Asian American populations in the U.S. [4]. As of 2017, about 2 million Korean American people, including 1 million immigrants born in Korea and 920,000 immigrants born outside Korea, resided in the United States [5]. The earlier influx of Korean immigrants to the U.S. was driven by mostly involuntary motivations such as poverty and being war victims or political refugees [6]. Korean immigrants significantly increased during the 1960s due to the removal of restrictions on Asian immigration to the U.S. [5] and the desire to find a stable life away from aspects of life in South Korea that included the low employment rate, military dictatorship, and insecure political climate [6]. More recent Korean immigrants are likely to have higher educational attainment and socioeconomic status (SES) than the U.S. general populations as well as other immigrant groups [5]. Korean immigrant adults often remain monolingual, speaking Korea language most of the time and socializing within co-ethnics including Korean religious groups, resulting in cultural and ethnic isolation [7] as well as psychological distress and malfunctioning [8,9].

Both the direct and indirect impacts of the COVID-19 pandemic on individual members of our society should be examined. One of the indirect effects is Korean immigrants’ experiences of increased racial discrimination due to the origin of the COVID-19 outbreak.

### 1.2. Increased Racism against Asian Populations in the U.S. Amid the COVID-19 Pandemic

On top of the common difficulties from the pandemic such as financial crisis, social isolation, and fear of disease, Asian populations in the U.S. have been experiencing an added layer of hardships since the beginning of the current pandemic [10]. As the first COVID-19 cases reported in the U.S. between January and February 2020 were identified from travelers arriving from China and their household contacts [11], Chinese and other East Asians were often seen as the origin and spreaders of the COVID-19 disease [12]. The term ‘Chinese virus’ or ‘Wuhan virus’ was continually used in early reports that blamed China as the epicenter of the COVID-19 pandemic [12]. The images of Asians wearing face masks were often accompanied by COVID-19 articles in major news media, which increased xenophobia and racism against Asian populations [13]. According to the Pew Research Center survey, about 39% of 278 Asian Americans in the U.S. have experienced more slurs or jokes than before the COVID-19 outbreak [14]. About the same portion of Asian Americans responded that, since the COVID-19 outbreak, people acted uncomfortable around them because they are Asian people [14]. Asian Americans continuously suffer from negative biases, overt racism, and racist attacks amid the COVID-19 pandemic. Finally, the Federal Bureau of Investiation (FBI) issued an official warning that hate crimes against Asian individuals in the U.S. might escalate since some Americans continue to associate the virus with China [15].

## 2. Literature Review

### 2.1. Racial Discrimination, Coping Mechanisms, and Mental Health

Racial discrimination has shown detrimental impacts on the psychological wellbeing of individual members from racial minority groups [16,17,18]. Specifically, minority individuals were likely to have low self-esteem and lack a sense of social or community belonging as a result of racial discrimination [19,20]. Several studies noted racial minorities’ experience of severe mental health problems, including The Diagnostic and Statistical Manual of Mental Disorders (DSM; latest edition: DSM-IV) disorders, depressive and anxious symptoms, and emotional problems [21,22,23]. According to Bernstein and colleagues, racial discrimination was a substantial factor in the mental health crisis of Korean immigrants in New York City [24].

Researchers have studied resilience as a critical coping resource in the face of inevitable life difficulties, referring to it as one’s ability to recover from or adjust to challenges such as trauma, adversity, or personal crises caused by life events [25]. Resilience is a dynamic process that is influenced by individual gender, age, cultural origin, and environmental or experiential variations, and is thus believed to be promoted through policy, intervention, and prevention [26]. Several studies have indicated resilience as a modifier of detrimental psychological wellbeing among immigrant populations. For instance, Bosma et al. said, resilience elements, such as “determination,” “commitment,” and “a will to overcome obstacles,” were modifying factors in overcoming life challenges (e.g., financial stability, racial discrimination, and immigration status) among Latino immigrant youth [27]. Bernstein et al. found high resilience buffered depressive symptoms of Korean immigrant adults who reported various traumatic life events, including stress related to acculturation and cultural shock [24]. In the study of West African immigrants’ stressors, including racial discrimination, and their mental health needs, participants portrayed the significance of resilience using culturally driven coping resources, such as community connectedness, religious faith, and African culture [28]. As a coping mechanism, resilience will function positively, decreasing one’s mental distress while enhancing psychological wellbeing in the presence of racial discrimination.

In an ethnically and racially diverse society, ethnic and racial identity (ERI) is an essential way of perceiving oneself for minority individuals [16]. Higher ERI is expected to promote positive effects on one’s psychological wellbeing by working as a coping strategy against racial discrimination [16,29]. Still, the protective role of ERI against racial discrimination has been debated [30]. Some studies have found positive roles of ERI in mental health enhancement against racial discrimination. For example, Mossaskowski found positive relationships between strong ethnic identity and reduced perceived racial/ethnic discrimination and decreased stress among Filipino Americans [31]. Other studies supported the countereffects of ERI on one’s psychological wellbeing during experiences of racism. One of the most recent studies on ERI noted higher ERI negatively influenced Asian immigrants’ perceived racial discrimination and psychological distress [16]. Yoo and Lee showed similar results, indicating negative relationships between higher ethnic identity and psychological wellbeing of Asian American college students when they were asked to respond to various imaginary racial discrimination scenarios. As ERI is a concept that is continually changing and is influenced by a variety of individual, social, and contextual factors [32], expanded exploration of the complicated role of ERI is essential.

Racial minorities benefit from social support greatly, especially with regard to emotional experiences and when they are dealing with perceived racial discrimination. Social support provides a sense of security and belongingness, which helps distract individuals from negative incidents [33]. Social support from an ethnic group also provides individuals with the understanding that racial discrimination is not only their problem but is a shared experience. Noh and Kaspar tested the mediating effect of ethnic social support between perceived racial discrimination and depression among the Korean immigrant population. Korean immigrants who were well connected with ethnic communities benefited from a problem-focused coping strategy rather than an emotional-focused coping style [34]. Social support from family members is another effective coping mechanism for racial minority populations. Social support among Asian Americans was found to have a moderating effect when this group experienced stress from racial discrimination [35]. Results indicate that perceived emotional support from family also buffered stress. Likewise, other studies also found that family support buffered the detrimental effects of racial discrimination with regard to the onset of major depressive disorders (MDD) [36,37].

### 2.2. Individuals’ Mental Health Status Amid the COVID-19 Pandemic

Although the impact of COVID-19 on individuals’ mental health is not yet fully assessed, there are some recent studies that have examined associations. In particular, a few studies examined the impact of perceived discrimination amid the COVID-19. Li et al. examined the effects of internalized stigmatization and social support on the mental health of Chinese respondents during the COVID-19 pandemic and found significant associations [38]. Another study examined the impact of perceived discrimination on mental distress [39]. The authors found that Non-Hispanic Blacks and Asians were more likely to perceive discrimination amid the pandemic than other racial groups. Also, the respondents who reported that they wear face masks were more likely to perceive discrimination, which, in turn, was associated with increased mental distress.

Other studies have explored the effects of other factors related to the COVID-19 pandemic on the individuals’ mental health status. One study unveiled the effects of the health factors related to the COVID-19 on the psychological distress in Spain [1]. Being a woman, being in a lower middle age group, having symptoms, and having contact with people who are confirmed with the COVID-19 predicted higher psychological distress. Consistently, Choi et al. found that being worried about getting infected from COVID-19, worrying about not having enough face masks, and not being able to work from home were associated with poorer mental health (e.g., depression and anxiety) in Hong Kong [40]. Furthermore, among American adults, exposure to social media and traditional media sources to learn about the COVID-19 was associated with a higher level of mental distress [41].

## 3. Materials and Methods

### 3.1. Procedure

Through purposive sampling, Korean immigrants above the age of 18 residing in the U.S. (including both foreign-born and U.S.-born) were invited to respond to a survey on Koreans’ wellbeing during the COVID-19 pandemic in the U.S. Invitations to participate were distributed by e-mails and posting on Korean immigrants’ online communities. Data were collected from 24 May 2020 to 14 June 2020. The study was conducted by following the Declaration of Helsinki [42] and was approved by the Ethics Committee of the University of North Florida (IRB #: 1289431-5). All participants gave their informed consent for inclusion before they participated in the study, and the data were stored anonymously.

### 3.2. Measurements

All the questions in the survey were developed by multicultural social science scholars who are both fluent in Korean and English languages. The surveys were available in both languages, and the participants were given the option to choose the language that they felt comfortable answering. Reliable and valid scales were used to measure the main variables, and the scales were translated from English to Korean. For the items that did not have any existing scales, researchers developed the questions in both languages. Afterwards, the authors back-translated all the questions, and outside reviewers, who are fluent in both languages, also checked the quality of the survey items. There were several reiterative steps to ensure that surveys in both languages were culturally competent and linguistically correct.

Psychological distress was measured using the Kessler Psychological Distress Scale (K10), which has high internal consistency with a Cronbach alpha reliability of 0.93 [43]. Participants described their psychological functioning over the past 30 days by responding to a set of 10 questions regarding psychological distress, anxiety, and depression. Some of the question examples include “About how often did you feel nervous?” and “About how often did you feel so sad that nothing could cheer you up?” A 5-point Likert-type scale, ranging from 1 (none of the time) to 5 (all the time), was used to measure the extent to which responders agree with each question. According to the National Comorbidity Survey [44], the difference between K6 and K10 is only the number of items. K6 is a shortened version that has four fewer items than the K10, but the two scales are equivalent in validity and reliability. The cut-off point for K10 is 24, and it is 14 for K6. The current study categorized the sample into 0 (low distress) vs. 1 (high distress) and used it as a binary variable for analysis.

Everyday discrimination measured individual experiences related to unfair treatment on a daily basis using a 9-item scale [45]. This scale has strong internal reliability, with a Cronbach’s alpha reliability of 0.88. Examples of the items include, “You are treated with less respect than other people” and “People act as if they think you are dishonest.” Participants demonstrate their experiences by answering on a 6-point Likert scale from 1 (never) to 6 (almost every day). A total score of the nine items, ranging from 9 to 45, was considered as a continuous variable.

Racial discrimination measured participants’ experiences of being disliked or unfairly treated because of their race using a 3-item scale [46]. The scale’s internal reliability is high, indicating Cronbach’s alpha reliability of 0.91. Respondents answer on a 4-point Likert scale from 0 (never) to 3 (often). A total score ranges from 0 to 9, and it was used as a continuous variable.

COVID-19 discrimination. Researchers developed the three items to measure the respondents’ perception of increased racial discrimination toward the Asian population since the beginning of the pandemic. Items include, “I believe that I am being discriminated against more frequently because of my race since the beginning of the COVID-19 pandemic” and “I believe that there is an increased negative perception toward the Asian population in my town since the beginning of the COVID-19 pandemic.” The responses can range from 1 (strongly disagree) to 5 (strongly agree), and the total score was used as a continuous variable.

Ethnic and Racial Identity (ERI). A set of four items measured respondents’ racial and ethnic identity, including items such as “How closely do you identify with other people who are of the same racial and ethnic descent as yourself?” and “If you could choose, how much time would you like to spend with other people who are of your same racial and ethnic group?” A 4-point Likert scale, ranging from 1 (not at all) to 4 (very much), is used to measure participants’ racial and ethnic identity. The variable was coded as a categorical variable indicating low, moderate, and high racial/ethnic identity on the basis of a previous study [47].

Resilience was measured by a 10-item scale, the Connor-Davidson resilience scale (CD-RISC-10) [48], which is a highly reliable and valid scale to measure an individual’s resilience. The scale is widely used in various cultures and is known to assess one’s resiliency in a highly valid way. Examples of the items include, “I can deal with whatever comes my way” and “I am not easily discouraged by failure.” The responses can range from 0 (Not true at all) to 4 (True nearly all the time). The Cronbach’s alpha was 0.85.

Social support is measured by using the three items from the Social Interaction Scale [49] and measured the following: (1) the extent to which the respondent can confine talk about worries to family, relatives, or friends; and (2) to what extent the respondent can rely on family, relatives, or friends to help with a serious problem. These items measure the respondents’ satisfaction of the social support that they think they will receive in a difficult situation. The responses can range from 1 (very dissatisfied) to 5 (very satisfied). The total score for social support (3–15) was used for analysis as a continuous variable.

Sociodemographic factors. Gender, age, marital status, years of education, household income, and employment status were controlled for analysis.

### 3.3. Data Analysis

All the analysis was conducted on SPSS 24.0. (SPSS Inc., Chicago, IL., USA). In order to account for missing values, the list-wise deletion was used if the remaining cases were large enough. A descriptive analysis was conducted in order to check the distribution of variables. Afterward, Artificial Neural Network (ANN) modeling was conducted in order to test the predictability of the three types of discriminations and three types of coping mechanisms on psychological distress. ANN is a preferred method for predictive data mining applications [50]. By mimicking the complex human brain activities, ANN predicts the relationships between variables in a powerful way. There are several advantages of using ANN, such as reducing statistical problems when analyzing non-linear multiple variables [51]. Furthermore, ANN can be used when there are associations between independent and dependent variables that are non-expressible via other methods. The multilayer perception (MLP) is a function of predictors that minimizes the prediction error of the output variables [52]. Therefore, ANN is the most suitable method, since the purpose of the current project was not limited to examine the association between study variables.

The first step is to assign the variables in the input, hidden, and output layers. Input layers contain the predictors, and the hidden layer has unobservable nodes or units. Based on some functions of the predictors, the value of each hidden unit is determined. The output layer contains the response or dependent variables. Again, each output unit is also determined by some function from the hidden unit [52]. During the ANN analysis, the dataset is randomly divided into training, test, and holdout sets. In the training set, data is used to train the neural network, and the testing sample is used to track errors so that overtraining (i.e., following random and meaningless patterns) is prevented. A holdout set is created to assess the final neural network. Since the holdout set is not used to create the model, it honestly estimates the model’s ability to predict [52]. After randomly selecting the three samples, data were scaled by Equation (1):

(1) $$X^{2}=\frac{X_{1}-\bar{X}}{\sigma_{x}},$$

where $X_{1}$ is the original value and $\bar{X}$ is the mean of a variable. $\sigma_{x}$ is the standard deviation of that variable, and $X^{2}$ is the new scaled value. After creating the three sets and scaling the data, the best transfer function was selected. It was selected among the neurons and a different transfer function for the output and hidden layers. Next, in a network, the impact of the number of hidden layers was assessed. In this study, the MLP feed-forward neural network was used and trained with the error back propagation algorithm. At the end of the modeling, the most straightforward network with the “highest sensitivity and specificity” was selected by the Receiver Operating Characteristic (ROC) curve [53]. In this model, the higher the sensitivity is, the lower the false-negative rate, and the higher the specificity, the lower the false-positive rate. The ROC curve measures how useful the model is. The bigger the Area Under the Curve (AUC) is, the more accurate the predictive result is [54]. More specifically, if the ROC curve is much higher than the diagonal reference line, it means that the given model is better at predicting the dependent variable. Lastly, the ANN determines the influence of factors and covariates as well.

## 4. Results

### 4.1. Subsection

Korean immigrants above the age of 18 who reside in the U.S. were recruited by an online survey. The final sample (*N* = 790) was recruited from 42 states. Table 1 shows the sample characteristics. In terms of psychological distress, almost half of the sample (49.4%) had a low level of psychological distress. The other half (49.2%) had a high level of psychological distress.

### 4.2. Artificial Neural Network Analysis

**Figure 1:**
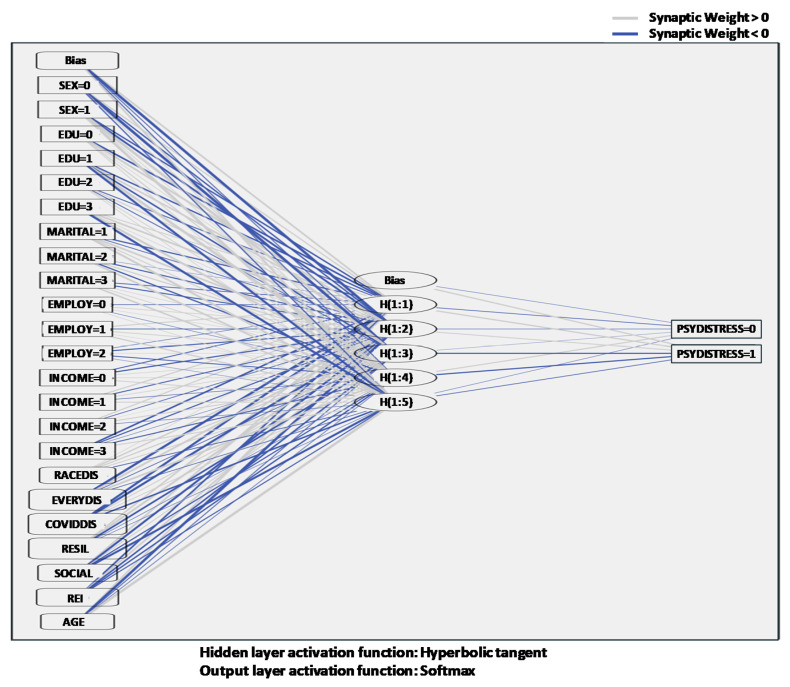
Network structure diagram with synaptic weights.

The ANN model examined the predictability of the three types of discrimination and three types of coping mechanisms on the respondents’ psychological distress. Of the entire dataset, 70.4% was randomly chosen to create a training sample. The other 11.6% was used as a testing sample, and the holdout sample used the last 18% of the dataset. The error for the training set was 25.3%, 24.7% for the testing set, and 36.1% for the holdout set, respectively. Figure 1 shows the model structure with synaptic weights. The thicker the lines are, the stronger the relationships between variables. In the input layer, there were five factors and seven covariates. Five factors include individuals’ sex, educational level, employment status, annual income, and marital status. Seven covariates are racial discrimination, everyday discrimination, discrimination during the COVID-19 pandemic, resilience, social support ERI, and age. In the hidden layer, there were five nodes and one bias unit. In the output layer, the respondents’ level of psychological distress was included as a dependent variable.

**Figure 2:**
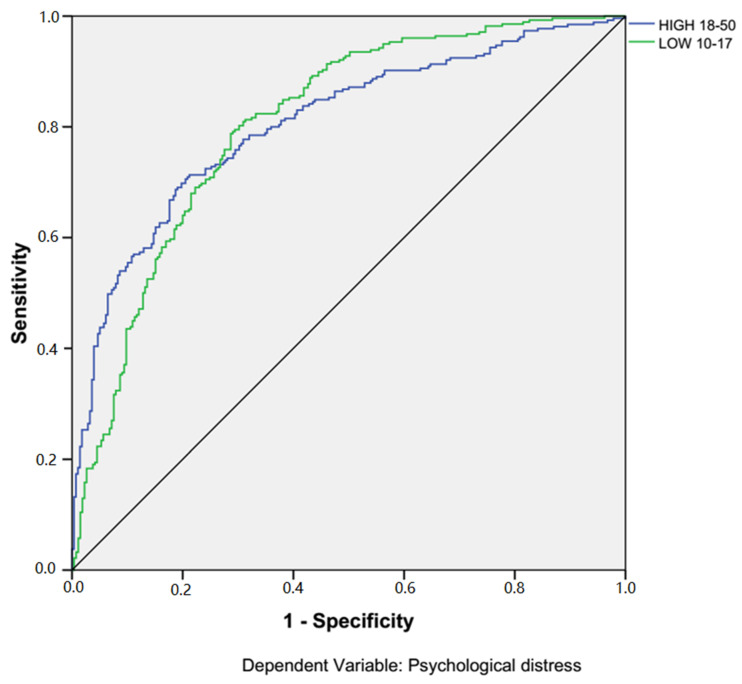
ROC curve.

The results from the sensitivity analysis are presented in Figure 2 and Table 2. Figure 2 shows the ROC curves for two categories, high vs. low, of the psychological distress level. The higher the ROC curves are from the diagonal line, which serves as the reference line, the better the model is [55]. Both of the ROC curves for two categories of the dependent variable in the model are far away from the diagonal line. Table 2 shows the Area Under the ROC Curve (AUC), which is 0.806 for both ROC curves. This means that the given neural network can predict a person with high vs. low psychological distress with sensitivity above 80.6%. AUC between 0.8 to 0.9 is considered an excellent model with the predictive ability [55]. Table 3 shows the importance of each independent variable in predicting the level of respondents’ psychological distress. A person’s resilience was the most important predictor (Importance 0.173; Normalized importance 100.0%). Experiences of everyday discrimination (Importance 0.144; Normalized importance 83.2%) was the second most important predicting variable. COVID-19 discrimination (Importance 0.144; Normalized importance 59.8%) and social support (Importance 0.095; Normalized importance 55.1%) were of moderate importance. Whereas, individuals’ age, sex, racial and ethnic identity, racial discrimination, and education level showed less importance in predicting the level of psychological distress. Finally, an individuals’ income level, employment status, and marital status had the least predictive abilities in the ANN model.

**Figure 3:**
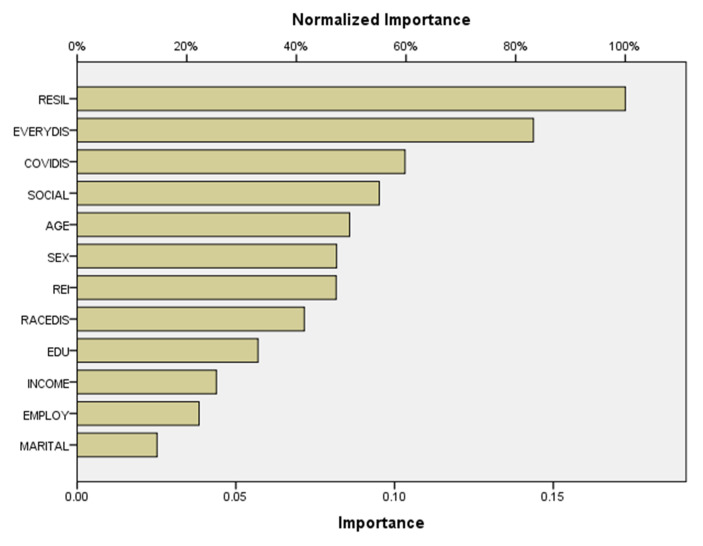
Normalized importance of independent variables.

Figure 3 illustrates the same information as Table 3 and shows clearly that resilience, everyday discrimination, COVID-19 discrimination, and social support are the most effective variables for predicting psychological distress. Some of the sociodemographic variables were of little importance, such as marital status, employment status, income, and educational level. A person’s age and sex were two demographic variables that impacted the dependent variable in a moderate way. In contrast, respondents’ perception of racial discrimination and racial/ethnic identity was of a moderate impact as well.

## 5. Discussion

In April 2020, 13.6% of U.S. adults reported having serious psychological distress symptoms, and this number is much higher than back in 2018 (3.9%) when there was no COVID-19 pandemic [56]. Korean immigrants in the current study showed a much higher serious psychological distress rate. Findings from our study showed that experiencing everyday discrimination and perceiving that the discrimination toward Asians has increased during the COVID-19 pandemic were both powerful predictors of the Korean immigrants’ psychological distress level. This is consistent with the previous studies showing the impact of perceived discrimination on individuals’ mental health [21,57,58]. Moreover, despite experiencing discrimination, individuals’ coping mechanisms, such as resilience and having social support, were important protective factors. In fact, resilience was the most powerful predictor in the model. This is also consistent with the previous studies on the role/influence of resilience on individuals when they are faced with difficulties and challenges [59,60].

Moreover, the fact that the current study’s findings did not show racial discrimination (on the 3-item scale by Vega [46]) as a significant predictor of individuals’ psychological distress is inconsistent with the previous literature. It is worth exploring how Korean immigrants perceive racial discrimination differently or similarly from other racial/ethnic groups. Furthermore, REI was not an important predictor of the Korean immigrants’ psychological distress in the study. In fact, this finding is consistent with the previous literature on REI and racial minority individuals’ mental health, which showed mixed findings [16,33]. Furthermore, individuals’ marital status, employment status, income, and education level were relatively unimportant predictors on the respondents’ psychological distress level. Individuals’ sex and age played more important roles than the abovementioned individual characteristics. In the end, the fact that the level of resilience was powerfully predicting individuals’ psychological distress is good news. It means that even if individuals experience discrimination, if they are resilient individuals, they are protected from the negative impact.

Findings from the current study call for the implementation of practice implications in a timely manner. We should not overlook Korean immigrants’ experiences of discrimination, which influences their psychological wellbeing. As our study observed, Korean immigrants have been exposed to high levels of everyday discrimination, especially amid COVID-19. The adverse effects of experiencing discrimination have been remarkably observed in our study. Fortunately, powerful results have been noted in resilience programs [61,62,63,64] for individuals who experienced social bias with poor mental health. Therefore, it would be very effective if such programs/interventions—Universal Resilience-Focused Intervention, Mindful Awareness and Resilience Skills, and A Whole Community Approach on Social Resilience—were employed for Korean immigrants who exhibit a high level of psychological distress.

Additionally, during the pandemic, when people need to keep social distancing, utilizing telehealth programs for implementation of resilience programs/treatments is strongly recommended. The telehealth program is to “promote the use of telehealth technologies for health care delivery, education, and health information services” [65]. This program has been effectively applied to patients living in rural or remote areas where people have difficulty accessing sufficient health care services or special clinics [65]. Several studies have already observed the effectiveness of telehealth programs for patents amid the COVID-19 pandemic [66,67]. During the current COVID-19 pandemic, many everyday routines of life and work have been quickly converted to the use of advanced technologies, such as videoconferencing and online delivery services [67]. Additionally, since social support was one of the crucial coping mechanisms for Korean immigrants in the sample, virtual social support via telehealth can be provided to the target population who might be devoid of the usual social support because of the pandemic.

In order to address the increased discrimination during the COVID-19, several experts stress the importance of having strong group solidarity among racial minority individuals to overcome their daily hardships [67,68]. The study by Chong and Rogers discovered that supporting each other within different racial/ethnic groups increases the awareness and empowerment of the ethnic minority people [68]. However, unfortunately, as opposed to Black people, Asian and Hispanic individuals have not built strong ethnic solidarity in the U.S. [68]. For Asian immigrants, building racial group solidarity is crucial for their wellbeing [68].

There are several limitations of the given study. First, the final sample of the study does not represent the entire Korean immigrant population in the U.S. since purposive sampling was utilized. The sample of the current study is from a relatively high socioeconomic group, and therefore, cannot represent the entire Korean immigrant experience in the U.S. amid the COVID-19 pandemic. Second, since the data collection took place during May–June 2020, without further data collection, we cannot know the long-term impact of discrimination amid the pandemic on the respondents’ psychological distress. Future studies can utilize random sampling strategies with multiple data collection in order to study the impact of discrimination over time from a nationally representative sample.

## 6. Conclusions

The current study made a meaningful contribution to the literature on COVID-19 and individuals’ mental health. Specifically, it is one of the first attempts to capture the effects of racial discrimination targeting the Asian population in the U.S., which has intensified since the beginning of the pandemic. By using ANN as a non-linear statistical data modeling tool, the given research unveiled the important predicting factors affecting Korean immigrants’ psychological distress in the U.S., especially during an unprecedented pandemic. The most important predicting factors were individuals’ level of resilience, experience of everyday discrimination, and perception that the racial discrimination toward Asian populations has intensified in the U.S. since the beginning of the pandemic. Findings from the study provide important implications for public health practitioners and community partners.

## Figures and Tables

**Table 1:** Characteristics of the Study Sample.

Mean or Percentage			
Variables	All Sample (*N* = 790)	Variables	All Sample (*N* = 790)
Dependent Variable		Covariates	
Psychological distress		Education	
Low	49.4%	High school diploma or less	7.9%
High	49.2%	Some level of college education	20.9%
Factors		Bachelor’s degree	34.4%
Racial discrimination (range: 0–9)		Graduate degree	36.8%
Mean: 2.87	Std.Dev: 2.06	Employment status	
Everyday discrimination (range: 9–54)		Employed full time	53.4%
Mean: 12.43	Std.Dev: 4.93	Employed part-time	14.3%
COVID-19 discrimination (range: 3–15)		Out of labor force	32.4%
Mean: 8.64	Std.Dev: 2.65	Marital status	
Resilience (range: 0–40)		Married	73.8%
Mean: 25.03	Std.Dev: 6.85	Never married	21.0%
Racial/Ethnic Identity (range: 0–16)		Widowed/separated/divorced	5.2%
Mean: 11.63	Std.Dev: 2.26	Household income	
Age (years)		<$34,999	21.6%
Min: 20	Mean: 42.74	$35,000–$49,999	13.3%
Max: 81	Std.Dev: 12.14	$50,000–$99,999	27.8%
Yrs in the U.S. (years)	Mean: 16	≥$100,000	27.8%
Range: 1–61	Std.Dev: 10.96	Sex	
U.S. Nativity		Male	40.3%
Foreign (South Korea)-born	90.9%	Female	59.7%
U.S.-born	1.9%		
Language Barrier			
Yes	27.3%		
No	72.7%		

**Table 2:** Area under the ROC curve (AUC).

Psychological Distress	AUC
Low 10–17	0.806
High 18–50	0.806

**Table 3:** Relative importance of independent variables.

Variable	Importance	Normalized Importance (%)
Resilience	0.173	100.0%
Everyday discrimination	0.144	83.2%
COVID-19 discrimination	0.103	59.8%
Social support	0.095	55.1%
Age	0.086	49.7%
Sex	0.082	47.3%
Racial Ethnic Identity	0.082	47.2%
Racial discrimination	0.072	41.4%
Education level	0.057	33.0%
Income	0.044	25.4%
Employment status	0.038	22.2%
Marital status	0.025	14.6%

## References

[1] Gomez-Salgado J., Andres-Villas M., Dominguez-Salas S., Diaz-Milanes D., Ruiz-Frutos C. (2020). Related Health Factors of Psychological Distress During the COVID-19 Pandemic in Spain. Int. J. Environ. Res. Public Health.

[2] World Health Organization Coronavirus Disease (COVID-19) Dashboard. https://covid19.who.int/.

[3] World Health Organization Global: United States of America. https://covid19.who.int/region/amro/country/us.

[4] Bureau U.S.C. Asian Alone or in Any Combination by Selected Groups. https://data.census.gov/cedsci/table?q=pakistani&g=&lastDisplayedRow=18&table=B02018&tid=ACSDT1Y2018.B02018&vintage=2018&mode.

[5] O’Connor A., Batalova J. (2019). Korean Immigrants in the United States.

[6] Boston University School of Theology: Boston Korean Diaspora Project. History of Korean Immigration to America, from 1903 to Present. http://sites.bu.edu/koreandiaspora/issues/history-of-korean-immigration-to-america-from-1903-to-present/#_ftn9.

[7] Yasui M., Kim T.Y., Choi Y. (2018). Culturally specific parent mental distress, parent-child relations and youth depression among Korean American families. J. Child Fam. Stud..

[8] Choi J., Miller A., Wilbur J. (2009). Acculturation and depressive symptoms in Korean immigrant women. J. Immigr. Minor. Health.

[9] Min J.W., Moon A., Lubben J.E. (2005). Determinants of psychological distress over time among older Korean immigrants and non-Hispanic white elders: Evidence from a two-wave panel study. Aging Ment. Health.

[10] Liu Y., Finch B.K., Brenneke S.G., Thomas K., Le P.D. (2020). Perceived discrimination and Mental Distress amid the COVID-19 pandemic: Evidence from the understanding America study. Am. J. Prev. Med..

[11] Schuchat A. (2020). Public Health Response to the Initiation and Spread of Pandemic COVID-19 in the United States, February 24–April 21,2020. MMWR Morb. Mortal. Wkly Rep..

[12] Marquardt A., Hansler J. (2020). US push to include ‘Wuhan virus’ language in G7 joint statement factures alliance. CNN Politics.

[13] Burton N. (2020). Why Asians in masks should not be the “face” of the coronavirus. Vox.

[14] Ruiz N.G., Horowitz J., Tamir C. (2020). Many Black and Asian Americans Say They Have Experienced Discrimination Amid the COVID-19 Outbreak.

[15] Margolin J. (2020). FBI warns of potential surge in hate crimes against Asian Americans amid coronavirus. ABC News.

[16] Choi S., Weng S., Park H., Hong J. (2020). Counter-Effects of Ethnic and Racial Identity (ERI) as a Buffer against Perceived Racial Discrimination among Asian Immigrants. Smith Coll. Stud. Soc. Work.

[17] Choi S., Weng S., Park H., Kim Y. (2020). Effects of Asian immigrants’ group membership in the association between perceived racial discrimination and psychological well-being: The interplay of immigrants’ generational status, age, and ethnic subgroup. J. Ethn. Cult. Divers. Soc. Work.

[18] Pieterse A.L., Todd N.R., Neville H.A., Carter R.T. (2011). Perceived racism and mental health among Black American. J. Couns. Psychol..

[19] Greene M.L., Way N., Pahl K. (2006). Trajectories of perceived adult and peer discrimination among Black, Latino, and Asian American adolescents: Patterns and psychological correelates. Dev. Psychol..

[20] Yoo H.C., Lee R.M. (2005). Ethnic identity and approach-type coping as moderators of the racial discrimination/well-being relation in Asian Americans. J. Couns. Psychol..

[21] Gee G.C., Spencer M., Chen J., Yip T., Takeuchi D.T. (2007). The association between self-reported racial discrimination and 12-month DSM-IV mental disorders among Asian Americans nationwide. Soc. Sci. Med..

[22] Lewis T.T., Cogburn C.D., Williams D.R. (2015). Self-reported experience of discrimination and health: Scientific advances, ongoing controversies, and emerging issues. Annu. Rev. Clin. Psychol..

[23] Li M. (2014). Discrimination and psychiatric disorder among Asian American immigrants: A national analysis by subgroups. J. Immigr. Minor. Health.

[24] Bernstein K., Park S.Y., Nokes K.M. (2017). Resilience and depressive symptoms among Korean Americans with history of traumatic life experience. Community Ment. Health J..

[25] Connor K.M., Davidson R.T. (2003). Development of new resilience scale: The Connor-Davidson Resilience Scale (CD-RISC). Depress. Anxiety.

[26] Masten A., Obradovic J. (2006). Competence and resilience in development. N. Y. Acad. Sci..

[27] Bosma L.M., Orozco L., Barriga C.C., Rosas-Lee M., Sieving R.E. (2019). Promoting resilience during adolescence: Voices of Latino youth and parents. Youth Soc..

[28] Akinsulure-Smith A.M. (2017). Resilience in the face of adversity: African immigrants’ mental health needs and the American Transition. J. Immigr. Refug. Stud..

[29] Phinney J.S. (1991). Ethnic identity and self-esteem: A review and integration. Hisp. J. Behav. Sci..

[30] Park I.J.K., Schwartz S.J., Lee R.M., Kim M. (2013). Perceived racial/ethnic discrimination and antisocial behaviors among Asian American college students: Testing the moderating roles of ethnic and American identity. Cult. Divers. Ethn. Minor. Psychol..

[31] Mossaskowaski K.N. (2003). Coping with perceived discrimination: Does ethnic identity protect mental health?. J. Health Soc. Behav..

[32] Tafoya S. Shades of belonging: Latinos and Racial Identity. https://files.eric.ed.gov/fulltext/ED486481.pdf.

[33] Brondolo E., Halen N.B., Pencille M., Beatty D., Contrada R.J. (2008). Coping with racism: A selective review of literature and a theoretical and methodological critique. J. Behav. Med..

[34] Noh S., Kaspar V. (2003). Perceived discrimination and depression: Moderating effects of coping, acculturation, and ethnic support. Am. J. Public Health.

[35] Mossakowski K.N., Zhang W. (2014). Does social support buffer the stress of discrimination and reduce psychological distress among Asian Americans?. Soc. Psychol. Q..

[36] Chae D.H., Lee S., Lincoln K.D., Ihara E.S. (2011). Discrimination, family relationships, and major depression among Asian Americans. J. Immigr. Minor. Health.

[37] Tummala-Narra P., Alegria M., Chen C.N. (2012). Perceived discrimination, acculturative stress, and depression among South Asians: Mixed findings. Asian Am. J. Psychol..

[38] Li J., Liang W., Yuan B., Zeng G. (2020). Internalized stiagatization, social support, and individual mental health problems in the public health crisis. Int. J. Environ. Res. Public Health.

[39] Choi E.P.H., Hui B.P.H., Wan E.Y.F. (2020). Depression and anxiety in Hong Kong during COVID-19. Int. J. Environ. Res. Public Health.

[40] Riehm K.E., Holingue C., Kalb L.G., Bennette D., Kapateyn A., Jiang Q., Veldhuis C., Johnson R.M., Fallin M.D., Kreuter F. (2020). Associations between media exposure and mental distress among U.S. adults at the beginning of the COVID-19 pandemic. Am. J. Prev. Med..

[41] Barrios Osuna I., Anido Escobar V., Morera Pérez M. (2016). Declaración de Helsinki: Cambios y exégesis. Rev.Cuba. Salud Publica.

[42] Kessler R.C., Andrews G., Colpe L.J., Hiripi E., Mroczek D.K., Normand S.-L.T., Walters E.E., Zaslavsky A.M. (2002). Short screening scales to monitor population prevalences and trends in non-specific psychological distress. Psychol. Med..

[43] K10 and K6 Scales. https://www.hcp.med.harvard.edu/ncs/k6_scales.php.

[44] Williams D.R., Yan Y., Jackson J.S., Anderson N.B. (1997). Racial differences in physical and mental health: Socio-economic status, stress and discrimination. J. Health Psychol..

[45] Vega W., Zimmerman R., Gil A., Warheit G., Apospori E. (1993). Acculturation Strain Theory: Its Application in Explaining Drug Use Behavior among Cuban and Other Hispanic Youth.

[46] Balsam K.F., Molina Y., Blayney J.A., Dillworth T., Zimmerman L., Kaysen D. (2015). Racial/ethnic differences in identity and mental health outcomes among young sexual minority women. Cult. Divers. Ethn. Minor. Psychol..

[47] Campbell-Sills L., Stein M.B. (2007). Psychometric analysis and refinement of the Connor-davidson resilience scale (CD-RISC): Validation of a 10-item measure of resilience. J. Trauma. Stress.

[48] Schuster T.L., Kessler R.C., Aseltine R.H. (1990). Supportive interactions, negative interactions, and depressed mood. Am. J. Community Psychol..

[49] Haykin S. (1998). Neural Networks: A Comprehensive Foundation.

[50] Wlodarczyk M., Dolinska-Zygmunt G. (2019). Searching for predictors of sense of quality of health: A study using neural networks on a sample of perimenopausal women. PLoS ONE.

[51] IBM SPSS Neural Networks 24. n.d. ftp://public.dhe.ibm.com/software/analytics/spss/documentation/statistics/24.0/en/client/Manuals/IBM_SPSS_Neural_Network.pdf.

[52] Allahyri E. (2019). Predicting elderly depression: An artificial neural network model. Iran. J. Psychiatry Behav. Sci..

[53] Ekelund S. ROC Curves-What Are They and How Are They Used?. https://acutecaretesting.org/en/articles/precision-recall-curves-what-are-they-and-how-are-they-used.

[54] Mandrekar J.N. (2010). Receiver operating characteristic curve in diagnostic test assessment. J. Thorac. Oncol..

[55] McGinty E.E., Presskreischer R., Han H., Barry C.L. (2020). Psychological Distress and Loneliness Reported by US Adults in 2018 and April 2020. JAMA.

[56] Dolezsar C.M., McGrath J.J., Herzig A.J.M., Miller S.B. (2014). Perceived racial discrimination and hypertension: A comprehensive systematic review. Health Psychol. Off. J. Div. Health Psychol. Am. Psychol. Assoc..

[57] Hwang W.-C., Goto S. (2008). The impact of perceived racial discrimination on the mental health of Asian American and Latino college students. Cult. Divers. Ment. Health.

[58] Zahran S., Peek L., Snodgrass J.G., Weiler S., Hempel L. (2011). Economics of disaster risk, social vulnerability, and mental health reselience. Risk Anal..

[59] Bonanno G. (2004). Loss, trauma, and human resilience: Have we underestimated the human capacity to thrive after extremely aversive events?. Am. Psychol..

[60] Black L., Van Agteren J., Isaiello M., Carey M., Faggotter R. (2018). Mental health interventions to build resilience. Aust. J. Emerg. Manag..

[61] Vo D.X., Locke J.J., Johnson A., Marshall S.K. (2015). The effectiveness of the mindful awareness and resilience skills for adolescents (MARS-A) intervention on adolescent mental health: A pilot clinical trial. J. Adolesc. Health.

[62] Dray J., Bowman J., Campbell E., Freund M., Hodder R., Wolfden L., Richards J., Leane C., Green S., Lecathelinais C. (2017). Effectiveness of a pragmatic school-based universal intervention targeting student resilience protective factors in reducing mental health problems in adolescents. J. Adolesc..

[63] Khanlou N., Wray R. (2014). A whole community approach toward child and youth resilience promotion: A review of resilience literature. Int. J. Ment. Health Addict..

[64] Health Resources & ServicesAdministration Telehealth Programs. https://www.hrsa.gov/rural-health/telehealth.

[65] Layfield E., Triantafillou V., Prasad A., Deng J., Shanti R.M., Newman J.G., Rajasekaran K. (2020). Telemedicine for head and neck ambulatory visits during COVID-19: Evaluating usability and patient satisfaction. J. Sci. Spec. Head Neck.

[66] Matheson B., Bohon C., Lock J. (2020). Family-based treatment via videoconference: Clinical recommendations for treatment providers during COVID-19 and beyond. Int. J. Eat. Disord..

[67] Chong D., Rogers R. (2005). Racial solidarity and political participation. Political Behav..

[68] Hoston W.T. (2009). Black solidarity and racial context: An exploration of the role of Black solidarity in U.S. cities. J. Black Stud..

